# European structural funds to finance healthcare in Italian regions

**DOI:** 10.3389/fpubh.2024.1361642

**Published:** 2024-10-04

**Authors:** Elisabetta A. Graps, Raffaele Lagravinese, Adriano Ruggiero

**Affiliations:** ^1^CREHTA, ARESS Regione Pugli, Bari, Italy; ^2^Department of Economics and Finance, University of Bari “A.Moro”, Bari, Italy

**Keywords:** European Union, structural funds, healthcare, investments, Italy

## Abstract

In this work, we explore the extensive utilization of European Union Structural Funds to enhance regional healthcare systems in Italy over the period 2014–2020. These funds serve as vital instruments for financing the construction, renovation, and modernization of healthcare facilities, as well as supporting medical research and technological innovation. They enable the implementation of disease prevention and health promotion programs and provide essential income support to vulnerable families through the European Social Fund. Our analysis found that EU funding allocated to “health-related” projects during the 2014–2020 programming period, amounts to just over 6.19 billion euros [5.1 billion financed by the European Regional Development Fund (ERDF) and just over 1 billion financed by the European Social Fund (ESF)], of which 65.88% is funded by European resources. These funds supported a total of 26,739 projects, with 22,529 funded by the ERDF, primarily focusing on infrastructure projects and the acquisition of new technologies in the healthcare sector. Meanwhile, the 4,210 projects funded by the ESF were dedicated to personnel training and public health policies in the regions. The European co-financing provided by the ERDF exceeded 63%, while for the ESF, the European share was approximately 77%. Notably, some regions have leveraged these funds to pioneer telemedicine and healthcare technologies, improving healthcare accessibility, especially in remote areas. However, regional disparities in fund allocation and utilization persist and coordinated strategies and cross-regional collaboration, emphasizing the sharing of best practices and the reinforcement of transnational projects, need to successfully address these calls and to promote convergence not only in economic but also in healthcare terms.

## 1 Introduction

European Structural Funds are long-term financing programs aimed at reducing economic and social disparities within the European Union by promoting regional development, economic and social cohesion, and competitiveness. Among the most well-known structural funds are the European Regional Development Fund (ERDF) and the European Social Fund (ESF), which cover almost 80% of the total structural funds.^1^ The ERDF aims to strengthen economic and social cohesion in the European Union by correcting imbalances between its regions. It supports investments in infrastructure, innovation, and sustainable development. The ESF, on the other hand, focuses on improving employment opportunities, education, and social inclusion. It funds initiatives to enhance skills, job prospects, and social cohesion, targeting disadvantaged groups to reduce inequalities. The European Structural and Investment Funds (ESIF) play a crucial role in catalyzing improvements in healthcare systems across member states. For instance, in Slovakia, during the 2007–2013 programming period, ESIF invested €237 million in Slovak hospitals, which led to statistically significant improvements in certain healthcare quality measures, such as the readmission rate within 30 days. This demonstrates that while ESIFs are not primarily intended for healthcare financing, they can significantly enhance healthcare infrastructure and service delivery (1).

European structural and investment funds are not specifically intended for healthcare financing, they can have an indirect impact on investments in this sector, depending on the policies and spending priorities of each region. These two funds can allocate a portion of EU resources to improve healthcare infrastructure, promote technological innovation in the medical field, train healthcare personnel, and enhance accessibility to healthcare services in different regions (2). They can also serve as a valuable source of funding for policies aimed at supplementing the income of people with disabilities who require assistance. Furthermore, the use of these funds has been associated with significant improvements in healthcare outcomes in various member states (3).

The allocation and specific use of structural funds, however, depend on the priorities of each Member State and the funding programs established in agreement with the European Commission. For instance, Hungary and Slovakia have implemented various measures to improve fund absorption and achieve strategic development goals, thereby enhancing public service infrastructure, including healthcare (4). In some cases, health-related projects funded by structural funds have led to reductions in health inequalities and better access to healthcare services, as noted by Neagu et al. (5). The integration of health equity into the structural funds’ framework has been an evolving process, adapting to the shifting priorities within the EU’s broader socio-economic goals.

While there is extensive literature on the impact of Cohesion funds on growth in various European regions [e.g., (6, 7)], there are still too few studies that have specifically analyzed the impact of structural funds on regional healthcare system investments.

For instance, Vukašina et al. (8) examined the impact of European Structural and Investment Funds (ESIFs) on regional development in the new EU member states, finding that an increase in ESIF per capita by 1% contributed to a GDP per capita increase of 0.0053–0.008%, depending on the model used. Similarly, Jánošková (9) analyzed the effect of ESIFs on economic indicators in Slovakia, concluding that there is a dependence between the implementation of ESIFs and both GDP per capita and unemployment rates, although the impact on GDP was relatively low.

Specifically focusing on healthcare, Murauskiene and Karanikolos (10) demonstrated how structural funds improved healthcare infrastructure in Lithuania, with clear results on clinical outcomes. The study also highlights that in Lithuania, a portion of European resources was invested in training programs for doctors, nurses, and other healthcare professionals. Similarly, Tijanić and Kersan-Škabić (11) have shown that investment in healthcare infrastructure through structural funds has had a positive impact on the quality of healthcare services and accessibility in Croatia. Medeiros (12) highlighted how Cohesion funds were used in Portuguese regions to improve hospitals, clinics, medical research facilities, and other healthcare services. Research on Central and Eastern European countries has shown that these funds significantly contributed to public health infrastructure improvements, economic growth, and overall regional development (4). Other works (13, 14) reported in their analysis a series of projects implemented in various Eastern European and Mediterranean regions where Cohesion funds were used to support medical and scientific research, promoting innovation and the development of new medical or pharmaceutical technologies, thus improving the quality of care provided. Furthermore, especially the ESF is explicitly designed to invest in disease prevention and health promotion programs to reduce long-term healthcare costs. According to McCarthy (3), the allocation of structural funds for health in new member states has been crucial for developing public health infrastructure and services. Healthcare investments can create new jobs, thus contributing to the economic and social development of the region and reducing health disparities (15, 16). Holecki et al. (17) described a series of public healthcare interventions funded by European structural funds in Visegrad countries (Poland, Czech Republic, Slovak Republic, and Hungary). Even more recently, Dubas-Jakóbczyk and Kozieł (18) emphasized how EU structural funds constituted an important source of infrastructural investments in Poland, especially for public hospitals. Moreover, Tijanić and Kersan-Škabić (11) highlight the role of structural funds in supporting healthcare policy reforms and promoting health equity in Croatian regions.

Despite Italian regions receiving a significant amount of structural funds, to our knowledge, there are currently no studies mapping healthcare investments in different Italian regions. This gap in the literature indicates a need for more comprehensive studies to understand the impact of structural funds on healthcare systems at the regional level in Italy, emphasizing the need for comprehensive analysis to optimize fund utilization. Therefore, the purpose of this work is to provide regional data on community funds aimed at infrastructure, technological, and training development in the healthcare field and, more broadly, on all policies that can impact public health policies in Italian regions.

In Italy, regional healthcare expenditure is allocated through a funding system known as the “*Accordo di Programma Quadro*” (APQ).^2^ This agreement establishes the criteria and modalities for the distribution of financial resources between the State and the regions for financing the National Health Service (SSN). The allocation of structural funds, on the other hand, is carried out through specific programs and projects activated at the regional level in agreement with the European Commission and therefore represents additional resources beyond the funding provided by the State.

## 2 Data

To analyze the Cohesion funds dedicated to healthcare investments, we consulted the *OpenCoesione database* (OpenCoesione—Home). The OpenCoesione Database is an Italian project aimed at providing transparency and accessibility to information regarding public funds allocated to cohesion projects in Italy. This database collects information from various sources, including public entities and institutions responsible.

for managing the funds. These data include information on funded projects, allocated resources, and the regions involved. The main goal of the platform is to enable citizens, researchers, non-governmental organizations, and other stakeholders to monitor and evaluate the use of public funds for cohesion projects. Projects that fall under multiple programming areas are reported in the dataset as a single record containing all the project-related information. The datasets are updated bi-monthly and published approximately 3 months after the reference date. The minimum reporting unit is the project. In our work, all information is associated with projects financed with a unique project identification code called CUP. The analysis presented here uses data available for the programming period 2014–2020, updated as of April 2023.

There is no unique dimension that identifies healthcare projects. Therefore, following the approach of Dubas-Jakóbczyk and Kozieł (18), we identified all projects as “health-related,” taking into account both the type of specific priority/task under which the project was realized (e.g., Priority: investments in health infrastructure) and the type of beneficiary (e.g., health services provider, Ministry of Health, Municipalities, etc.).

The projects were aggregated based on the funding source. In order to better categorize the investments, we analyzed projects financed by the ERDF, which mainly include projects for the infrastructure and technological enhancement of regional healthcare systems (both public and private), and projects financed by the ESF, which are related to professional training of medical and nursing staff, health improvement policies in workplaces, educational programs from childhood to old age, income support programs for non-self-sufficient individuals, and overall interventions for the improvement of public health.

## 3 Descriptive analysis of ERDF and ESF funds

The aggregate analysis of various EU funds allocated to “health-related” projects during the 2014–2020 programming period amounts to just over 6.19 billion euros (5.1 billion funded by the ERDF and just over 1 billion funded by the ESF), of which 65.88% is financed by European resources (Table 1). These resources have funded a total of 26,739 projects, with 22,529 funded by the ERDF, primarily focusing on infrastructure projects and the acquisition of new technologies in the healthcare field. Meanwhile, the 4,210 projects funded by the ESF were dedicated to personnel training and public health policies in the regions. European co-financing provided by the ERDF exceeded 63%, while for the ESF, the European share was approximately 77%.

**Table 1:** Analysis of European co-financing: comparative analysis of co-financing devolved by the European Regional Development Fund (ERDF) and the European Social Fund (ESF) for project implementation at the national level.

Funds	Projects	Total investments	EU co-funded	EU/Total investments (%)
ERDF	22,529	5,177,882,760.63 €	3,293,685,539.48 €	63.61%
EFS	4,210	1,020,848,264.98 €	790,010,665.74 €	77.39%
Total	26,739	6,198,731,025.61 €	4,083,696,205.22 €	65.88%

**Figure 1:**
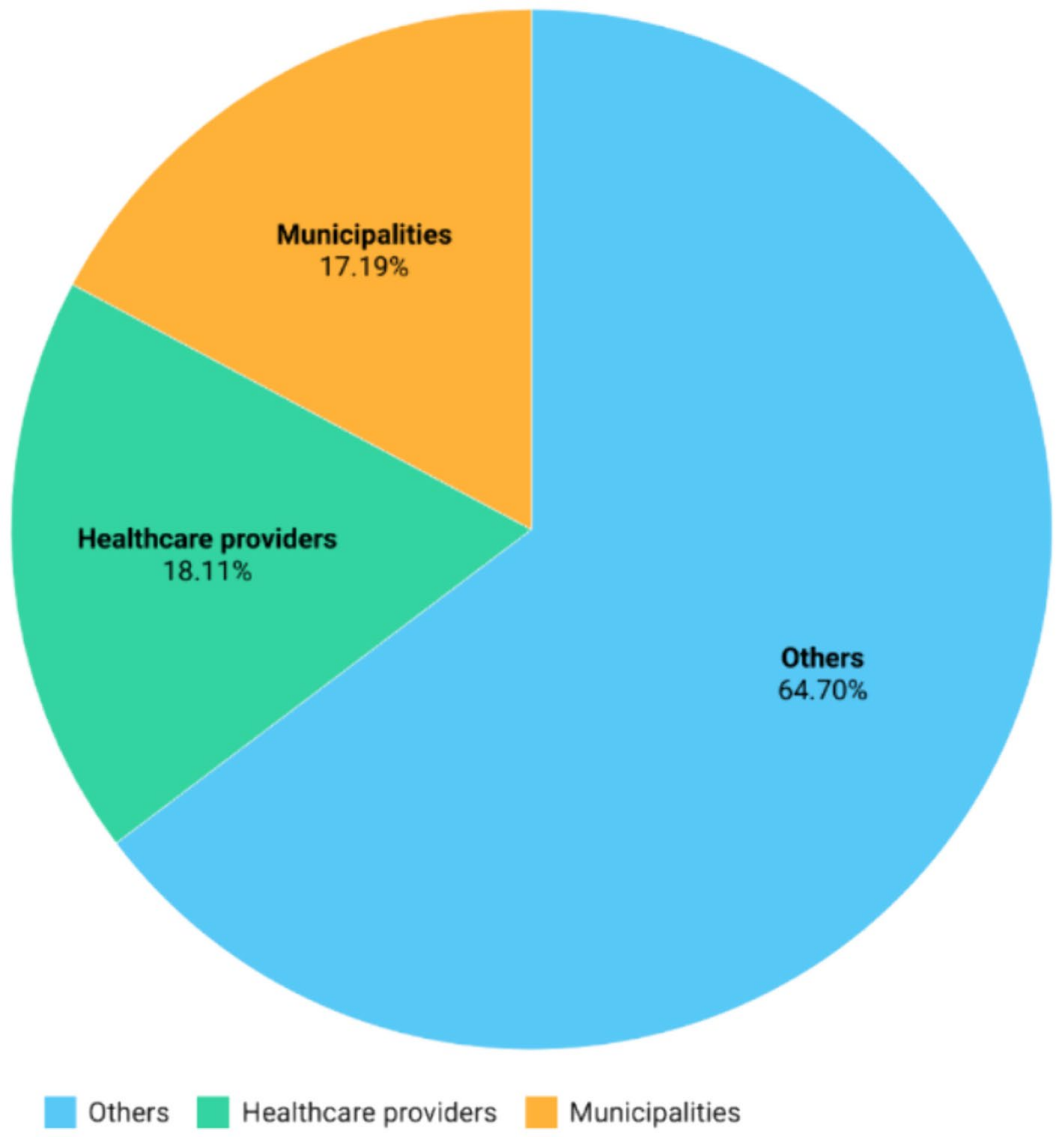
Total funding toward different categories of beneficiaries: relative total funding including European co-financed devolved by the European Regional Development Fund (ERDF) to the various categories of beneficiaries.

Regarding the recipients of ERDF funds (Figure 1), 17.19% of the total resources, including both funds from European sources and state financing, were allocated to municipalities. The 18.11% was directed to public or private entities that directly provide healthcare services (healthcare providers), while the remaining portion, amounting to 64.70%, was allocated to others. This category encompasses various other healthcare service providers such as private companies, educational institutions, non-governmental organizations indirectly providing health and social services.

Looking at the regional distribution of ERDF funds reported in Table 2, it is evident that there is significant heterogeneity among regions both in terms of total resources and the number of funded projects. Specifically, the Southern regions of Puglia and Campania received over 983 million euros (with 79% financed by EU contributions), and over 974 million euros (with 68% from EU funding) for investments in infrastructure and new technologies, respectively.

**Table 2:** Analysis of European co-financing (ERDF): analysis of co-financing devolved by the European Regional Development Fund (ERDF) for project implementation at the regional level.

REGIONS	Total Investment	EU Co-funded	EU co-funded/Total investment (%)
Abruzzo	127,680,991.62 €	66,609,723.20 €	52.17%
Basilicata	93,017,215.26 €	66,640,488.47 €	71.64%
Calabria	171,658,999.34 €	127,497,974.49 €	74.27%
Campania	974,607,920.52 €	664,532,826.32 €	68.18%
Emilia-Romagna	247,500,529.30 €	121,729,848.83 €	49.18%
Friuli-Venezia Giulia	48,792,115.62 €	24,413,885.52 €	50.04%
Lazio	429,190,719.48 €	336,960,248.04 €	78.51%
Liguria	43,190,080.94 €	17,187,104.04 €	39.79%
Lombardia	214,166,035.42 €	109,194,320.22 €	50.99%
Marche	110,111,052.49 €	55,522,863.20 €	50.42%
Molise	30,371,873.43 €	23,627,746.99 €	77.79%
Piemonte	221,195,756.17 €	101,945,566.81 €	46.09%
Puglia	983,548,324.63 €	772,864,338.66 €	78.58%
Sardegna	154,249,168.66 €	74,349,275.52 €	48.20%
Sicilia	569,582,527.76 €	355,998,716.14 €	62.50%
Toscana	596,741,552.83 €	264,730,912.29 €	44.36%
Trentino-Alto Adige	17,133,317.99 €	8,544,826.86 €	49.87%
Umbria	29,894,944.96 €	14,798,362.40 €	49.50%
Valle D’Aosta	24,887,189.13 €	7,720,586.56 €	31.02%
Veneto	170,262,338.22 €	78,815,924.92 €	46.29%
Total	5,257,782,653.77 €	3,293,685,539.48 €	62.64%

Source: authors’ elaboration on OpenCoesione data (April 2023).

**Figure 2:**
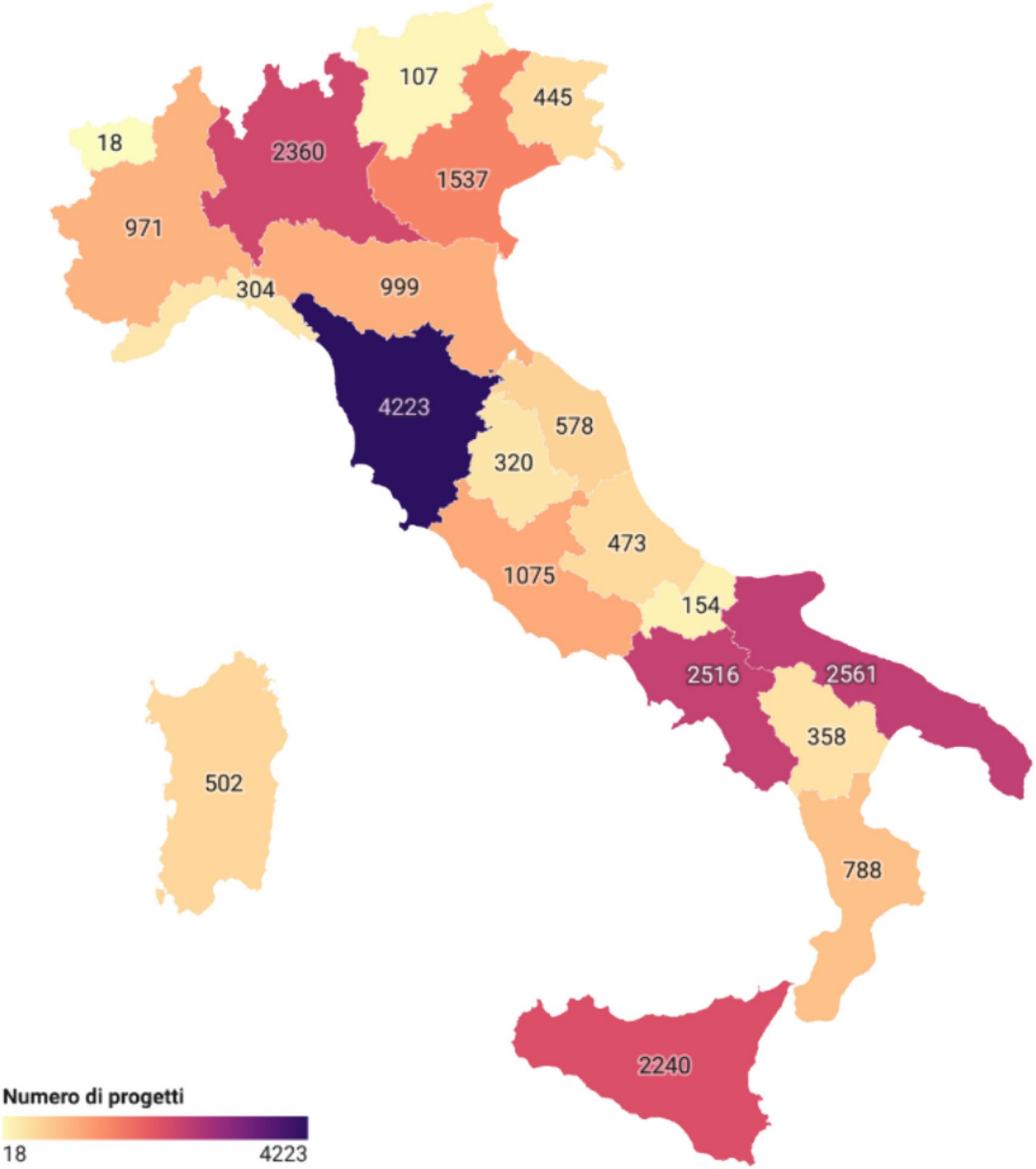
Number of projects co-financed by the ERDF: number of regional projects co-financed by the European Regional Development Fund (ERDF).

When considering the number of projects (Figure 2), Tuscany leads with 4,223 projects (18.74% of the total), followed by Puglia with 2,561 projects (11.37%) and Campania with 2,516 projects (11.17%). It is not surprising that the highest resources were allocated to the more populous Southern regions, as both Puglia and Campania are part of the Convergence regions, which receive more funding. Many of these investments were directed toward the modernization of healthcare facilities, technological innovation, the implementation of new healthcare monitoring methods such as telemedicine, and the renewal of diagnostic equipment.

The European Social Fund (ESF) financing allocated to “health-related” policies amounts to just over 1 billion euros in total, of which 790 million euros represent European co-financing. The territorial distribution of funds and projects (Table 3) essentially confirms what was observed for the ERDF. In this case as well, the region of Puglia stands out for a significant amount of resources allocated to education and training for children and older people, with over 299.5 million euros in total investment, of which 86.85%, equivalent to more than 260 million euros, comes from European Union contributions.

**Table 3:** Total funding toward different categories of beneficiaries: relative total funding including European co-financed devolved by the European Social Fund (ESF).

Regions	Total investment	EU co-funded	EU co-funded/Total investment (%)
Abruzzo	14,643,590.96 €	4,076,779.33 €	27.84%
Basilicata	18,654,741.76 €	15,094,824.32 €	80.92%
Calabria	31,257,738.62 €	23,686,430.52 €	75.78%
Campania	115,933,816.04 €	89,022,255.25 €	76.79%
Emilia-Romagna	73,113,201.96 €	38,751,737.44 €	53.00%
Friuli-Venezia Giulia	2,895,148.38 €	1,763,429.78 €	60.91%
Lazio	37,308,379.18 €	20,806,036.86 €	55.77%
Liguria	23,748,197.84 €	15,544,150.35 €	65.45%
Lombardia	26,154,047.10 €	16,378,731.16 €	62.62%
Marche	46,855,504.45 €	23,958,642.44 €	51.13%
Molise	1,907,841.92 €	1,665,503.82 €	87.30%
Piemonte	8,482,269.05 €	5,267,414.42 €	62.10%
Puglia	299,500,703.80 €	260,105,562.19 €	86.85%
Sardegna	15,957,580.89 €	8,965,056.72 €	56.18%
Sicilia	120,984,216.68 €	106,294,336.10 €	87.86%
Toscana	123,405,291.16 €	121,584,079.05 €	98.52%
Trentino-Alto Adige	9,014,072.24 €	4,511,363.31 €	50.05%
Umbria	31,691,236.53 €	16,228,039.88 €	51.21%
Valle D’Aosta	1,994,087.31 €	997,043.66 €	50.00%
Veneto	24,339,123.28 €	15,309,249.14 €	62.90%
Total	1,027,840,789.15 €	790,010,665.74 €	76.86%

Source: authors’ elaboration on OpenCoesione data (April 2023).

**Figure 3:**
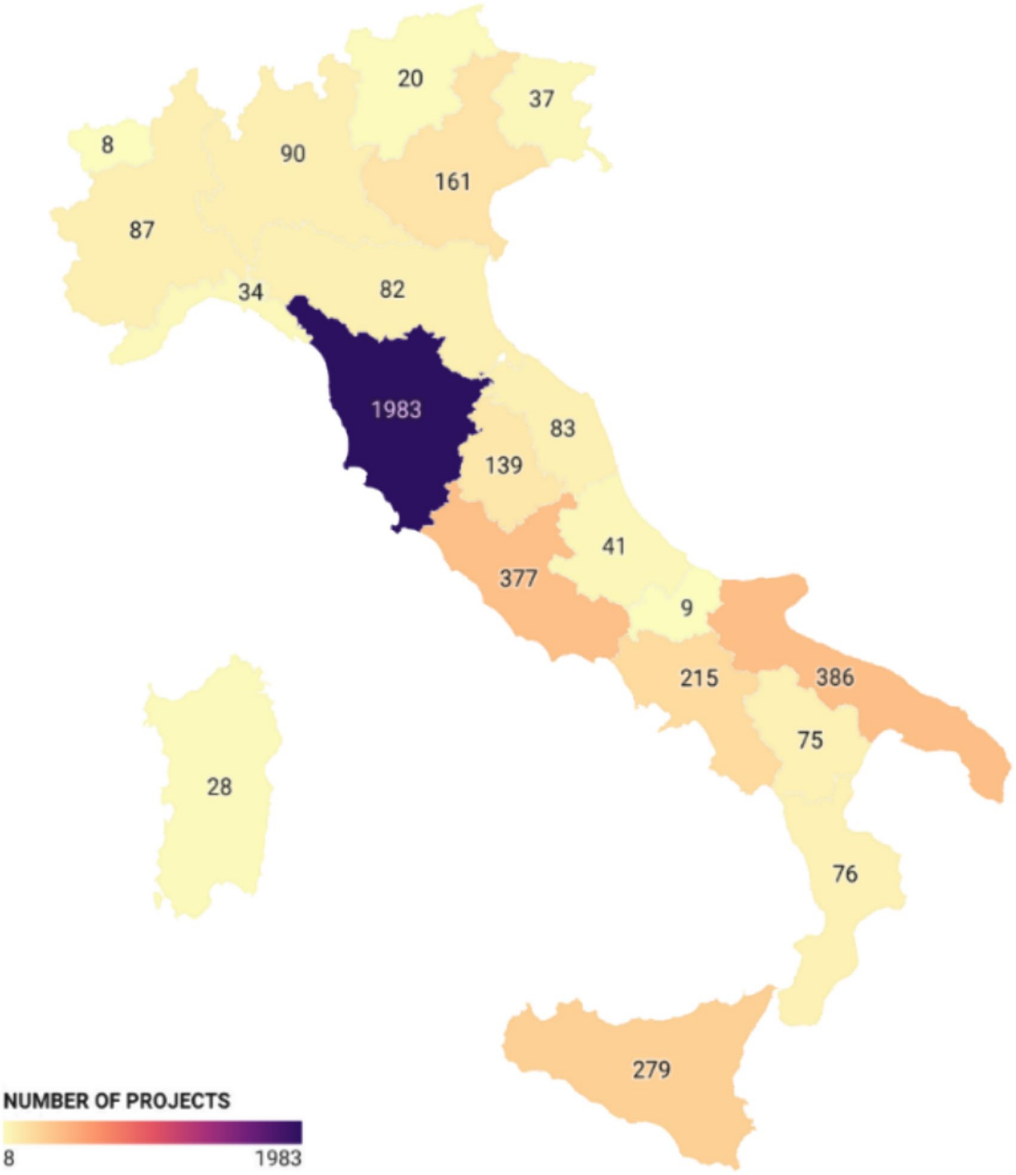
Number of projects co-financed by the ESF: number of regional projects co-financed by the European Social Fund (ESF).

Looking at the number of projects (Figure 3), Tuscany is in the lead with 1,983 projects, followed by Puglia with 386 projects and Lazio with 377 projects. In these regions, most of the projects were initiated by municipalities to finance service vouchers for non-self-sufficient individuals. This is an income support measure managed at the municipal level to assist financially disadvantaged individuals who are ill. Finally, a smaller portion of the funds was directed toward enhancing the training of personnel working in the regions.

## 4 Structural funds for COVID-19 response

The 2014–2020 programming period unfortunately coincided with the COVID-19 pandemic emergency. As a result, a significant portion of the regional resources was used to address the crisis. According to the 2023 Annual Summary Report by the European Commission, substantial efforts were made across Europe to mitigate the impact of COVID-19 using European funds (ERDF and ESF). In the Opencoesione database, there is a subsection dedicated to analyzing funds used to combat the COVID-19 emergency, utilizing European funds (ERDF and ESF). The total resources attributed to individual regions amount to 1.7 billion euros, to which additional funding from national projects and other projects that affected multiple regions should be added, bringing the overall amount to 3.2 billion euros. A substantial portion of these resources was also allocated to provide low-income families and workers with technological equipment (e.g., computers, tablets, etc.) to enable remote learning.

Looking at the territorial distribution (Table 4), it is evident that the majority of the resources were allocated to Campania (over 339 million euros with 571 projects), followed by Lazio (over 222 million euros with 446 projects), and Tuscany (over 213 million euros with 4,093 projects). Puglia allocated resources from both the ERDF and ESF, totaling more than 204 million euros across 387 projects.

**Table 4:** Regions funded by ERDF and ESF to counter the pandemic.

Regions	Total investment	EU co-funded	EU co-funded/Total investment (%)
Abruzzo	51.650.533,76 €	35.016.402,67 €	67.79%
Basilicata	15.707.159,24 €	14.083.281,23 €	89.66%
Calabria	5.067.620,87 €	3.308.906,53 €	65.30%
Campania	339.596.236,01 €	246.944.320,47 €	72.72%
Emilia-Romagna	90.064.138,51 €	44.963.214,40 €	49.92%
Friuli-Venezia Giulia	2.261.897,91 €	1.667.547,25 €	73.72%
Lazio	222.615.461,76 €	211.657.943,75 €	95.08%
Liguria	10.315.189,94 €	5.619.594,17 €	54.48%
Lombardia	23.493.635,96 €	15.817.375,96 €	67.33%
Marche	28.126.935,91 €	14.868.138,30 €	52.86%
Molise	24.906.606,56 €	17.583.533,06 €	70.60%
Piemonte	136.599.950,18 €	69.750.352,73 €	51.06%
Puglia	204.810.318,84 €	182.814.605,88 €	89.26%
Sardegna	41.782.477,78 €	21.338.424,72 €	51.07%
Sicilia	173.332.065,40 €	140.228.941,81 €	80.90%
Toscana	213.795.539,70 €	145.281.239,96 €	67.95%
Trentino - Alto Adige	11.329.170,23 €	5.914.585,06 €	52.21%
Umbria	18.175.928,89 €	9.096.273,11 €	50.05%
Valle D’Aosta	9.089.670,40 €	4.470.615,33 €	49.18%
Veneto	96.997.770,74 €	44.773.771,58 €	46.16%
Total	1.719.718.308,59 €	1.235.199.067,97 €	71.83%

Source: authors’ elaboration on OpenCoesione data (April 2023).

## 5 Discussion

As our analysis has attempted to highlight, European Union Structural Funds have been widely used to enhance regional healthcare through a range of targeted strategies and projects. These funds can be seen as an additional investment tool to finance the construction, renovation, or modernization of healthcare facilities such as hospitals, diagnostic centers, clinics, and long-term care facilities. Over the years, these resources have helped ensure that facilities are equipped with state-of-the-art equipment and technologies.

The funds can finance medical research and technological innovation in the healthcare sector, fostering the development of cutting-edge diagnostic and therapeutic solutions. They can be used to implement disease prevention and health promotion programs at the regional level. This may include awareness campaigns, vaccination programs, and initiatives promoting a healthy lifestyle. The European Social Fund is a crucial tool for supporting care for non-self-sufficient individuals and represents a fundamental form of income support for less affluent families.

As we have emphasized, these funds can accompany substantial changes in healthcare and assistance processes, as seen in some regions where telemedicine and healthcare technologies have been implemented, enabling remote diagnosis and treatment and improving access to healthcare services, especially in remote areas. Structural funds have also been used to develop emergency preparedness and management programs for healthcare crises such as pandemics or natural disasters, enhancing the capacity of regional healthcare facilities to respond effectively to critical situations.

Our analysis found that EU funding allocated to “health-related” projects during the 2014–2020 programming period amounted to just over 6.19 billion euros. This included 5.1 billion euros financed by the ERDF and just over 1 billion euros financed by the ESF. These funds supported a total of 26,739 projects, with 22,529 funded by the ERDF, primarily focusing on infrastructure projects and the acquisition of new technologies in the healthcare sector. Meanwhile, the 4,210 projects funded by the ESF were dedicated to personnel training and public health policies in the regions. The European co-financing provided by the ERDF exceeded 63%, while for the ESF, the European share was approximately 77%.

Regarding the recipients of ERDF funds, 17.19% of the total resources were allocated to municipalities, 18.11% to healthcare providers, and the remaining 64.70% to various other healthcare service providers, including private companies and educational institutions. The regional distribution of ERDF funds revealed significant heterogeneity, with Southern regions like Puglia and Campania receiving the highest investments. Puglia received over 983 million euros (79% financed by EU contributions), and Campania received over 974 million euros (68% from EU funding). Tuscany led in the number of projects with 4,223, followed by Puglia with 2,561 and Campania with 2,516.

For the ESF, the regional distribution also confirmed the prominence of regions like Puglia, which received significant resources for education and training for children and older persons, totaling over 299.5 million euros with 86.85% coming from EU contributions. Tuscany again led in the number of projects with 1,983, followed by Puglia with 386 and Lazio with 377.

Numerical comparisons from the European Structural and Investment Funds data reveal that the EU’s cohesion policy investments in health have been substantial. For example, the Coronavirus Response Investment Initiatives (CRII/CRII+) in 2020 increased health allocations to EUR 16.8 billion from over EUR 10 billion in 2019, demonstrating a significant rise in funding to support healthcare in response to the COVID-19 pandemic. The ERDF funded projects like the EUR 71 million investment in Bulgaria to improve emergency healthcare services, providing modern infrastructure and 400 new ambulances. Additionally, cross-border cooperation projects between Lithuania and Poland enhanced emergency response times and promoted the EU-wide 112 emergency number.

Our analysis confirms that European Structural Funds have significantly contributed to the enhancement of regional healthcare systems through various targeted strategies and projects. The case of Slovakia, where ESIF investments resulted in measurable improvements in hospital readmission rates, underscores the potential of these funds to address specific healthcare challenges. However, as noted by Fidrmuc et al. (1), the primary challenge remains the efficient use of available resources rather than the mere availability of funds. This aligns with broader findings in the literature that emphasize the need for systemic efficiency improvements to maximize the benefits of financial investments in healthcare.

In conclusion, regional analysis has shown significant heterogeneity in the use of funds and their distribution at the territorial level. The territorial distribution is greatly influenced by the ability of regional authorities to activate these resources through fundable projects. To address this, a coordinated approach and unified healthcare service development strategy should be pursued, requiring greater collaboration and coordination among the various Italian and European regions. In the coming years, the dissemination of best practices should be enhanced, allowing for learning from effective approaches adopted by other regions and their implementation at the local level. Furthermore, transnational projects should be strengthened to finance research projects, the development of networks of excellence, or training programs that could contribute to improving healthcare services and ensuring a more equitable distribution at the regional level. This approach could benefit not only wealthier regions but also deprived regions, promoting convergence not only in economic but also in healthcare terms.

## Data Availability

Publicly available datasets were analyzed in this study. This data can be found at: https://opencoesione.gov.it/it/.

## Footnotes

^1^The Partnership Agreement between the EU and Italy, adopted on October 29, 2014, and subsequently revised in February 2018, outlines the strategic framework and the selection of thematic objectives for which investments funded by the European Structural and Investment Funds (ESIF) are focused. In total, it represents approximately 44.8 billion euros of EU resources allocated to Italy, with over 36 billion euros specifically earmarked for cohesion policy, through the European Regional Development Fund (ERDF) and the European Social Fund (ESF).

^2^The APQ was introduced with Law No. 67 on March 28, 1988.
